# Targeting PINK1 and MQC in brain tumors

**DOI:** 10.18632/oncotarget.1980

**Published:** 2014-05-17

**Authors:** Kyu-Sun Lee, Bingwei Lu

**Affiliations:** Department of Pathology, Stanford University School of Medicine, Stanford, CA 94304, USA

Glioblastoma multiforme (GBM) is one of the most common brain tumors with very poor prognosis, and one of the most lethal tumors due to its highly invasive nature, high recurrence, and resistance to multimodal cancer therapy. GBM contains small subpopulations of cancer therapy-resistant tumor-initiating cells (also referred to as cancer-stem cells; CSCs) that share functional properties, such as self-renewal and capability of multi-lineage differentiation, with normal neural stem cells (NSCs). CSCs are believed to play a central role in the initiation and development of various tumors, as well as the recurrence of malignant tumors after conventional therapy. The identification of CSCs in various tumors has opened exciting new therapeutic opportunities. However, the molecular pathways maintaining the stem cell-like properties and tumorigenic behavior of the CSCs are poorly defined.

Cancer cells are known to undergo active glycolysis even in the presence of sufficient oxygen, which would promote more efficient energy production through mitochondrial oxidative phosphorylation (OXPHOS). This phenomenon was referred to as the Warburg effect. Although it was initially assumed that cancer cells are defective in OXPHOS, later studies have found that some cancer cells, including CSCs, are more active in OXPHOS than normal cells. Further, OXPHOS has been exploited for cancer treatment. Very little is known about the regulation and function of OXPHOS in CSCs.

The Notch signaling pathway plays a critical role in cell fate determination in NSCs and CSCs. Aberrant Notch activation is found in a wide range of tumors, including leukemia, breast cancer, and GBM. Notch mediates short-range cell-to-cell communication through interaction with ligands presented by neighboring cells. Notch consists of a ligand-binding extracellular domain, a transmembrane domain, and an intracellular domain (NICD) with transcriptional activity. In canonical Notch signaling, ligand-receptor interaction leads to sequential proteolytic cleavages and the translocation of cleaved NICD to the nucleus, where it regulates Notch-dependent transcription through interacting with transcription factor(s). Although the canonical Notch signaling pathway and its core components have been heavily studied, Notch can also act non-canonically to exert its biological effects by interacting with cytoplasmic components. Here, we discuss the emerging mechanisms and roles of non-canonical Notch signaling in preserving NSCs and CSCs through regulation of mitochondrial function.

*Drosophila* neuroblasts (NBs) offer an excellent model for recapitulating key aspects of stem cell biology underpinning stem cell maintenance and brain tumorigenesis. NBs are similar to mammalian NSCs in lineage hierarchy, including the presence of transit-amplifying intermediate progenitors (IPs). Inhibition of Notch signaling leads to NB loss, whereas enhanced Notch signaling causes the dedifferentiation of IPs into ectopic NB-like cells, leading to a brain tumor phenotype. In previous studies, canonical Notch signaling was shown to regulate the transcription of Notch target gene, Myc, whose regulation of cell growth is critical for the maintenance of NBs and CSC-like stem cells. However, gain-of-function studies indicated that canonical Notch signaling is insufficient to account for the Notch activation-induced brain tumor phenotype.

In a recent study, we searched for additional Notch signaling mechanisms important for brain tumorigenesis (Lee et al 2013). We found that the activity of the mechanistic target of rapamycin complex 2 (mTORC2)-AKT pathway was significantly elevated both in Notch-induced brain tumor model in *Drosophila* and in human GBM cells. Our genetic studies suggest that activation of mTORC2-AKT signaling by Notch is independent of the canonical pathway, indicating the presence of a non-canonical pathway. We previously observed strong genetic interaction between mTORC2 and PTEN-induced kinase 1 (PINK1) in maintaining mitochondrial integrity and function. Loss-of-function mutations in *PINK1* are associated with mitochondrial defects and cause degeneration of dopaminergic neurons, a cardinal feature of Parkinson's disease (PD), whereas increased PINK1 expression was found in different cancers, suggesting the involvement of PINK1 in both neurodegeneration and tumorigenesis. We found that Notch and PINK1 proteins are enriched in the mitochondrial fraction of human patient-derived GBM CSCs, and that they physically interact, suggesting a novel function of Notch in mitochondria.

Functionally, we found that *Drosophila PINK1* mutants exhibited defects in normal NB maintenance. Importantly, RNAi-mediated PINK1 inhibition blocked the activation of mTORC2 and abrogated the preservation of CSCs in both Notch-induced *Drosophila* brain tumor model and human GBM culture, supporting a critical role of PINK1 in NSC and CSC regulation. Moreover, like *PINK1* mutant, *Notch* mutant animals displayed defects in mitochondrial shape, ATP production, respiratory chain complex assembly, and DA neuron maintenance, supporting the potential involvement of Notch in PD. Further supporting the involvement of mitochondria in Notch-associated brain tumorigenesis, genetic perturbation of key mitochondrial processes, including OXPHOS (complex-I), biogenesis (PGC-1α), and fission (Drp1), also rescued Notch-induced brain tumor formation, and pharmacological inhibition of complex-I or Drp1 significantly blocked the maintenance of human GBM cells.

Metabolic remodeling is increasingly being recognized as a key feature of cancer cells. However, the pathogenic role of mitochondria in tumorigenesis remains unclear. Our recent studies revealed that Notch interacts with PINK1 to influence mitochondrial function, inducing the activation of mTORC2/AKT signaling in a non-canonical fashion. Moreover, PINK1, a key regulator of mitochondrial quality control (MQC), is essential for the maintenance of CSCs in both fly and humans. Thus, another important implication of this study is that PINK1 and the entire MQC signaling pathway contribute to CSC maintenance and represent novel targets for cancer intervention.
